# The Long-Term Effect of Maternal Iron Levels in the Second Trimester on Mild Thinness among Preschoolers: The Modifying Effect of Small for Gestational Age

**DOI:** 10.3390/nu15183939

**Published:** 2023-09-11

**Authors:** Kai-Wen Wang, Zheng-Jia Ling, Zhi Yuan, Jin Zhang, Song-Jia Yi, Yong-Wei Xiong, Wei Chang, Zhi-Jing Lin, Hua-Long Zhu, Lan Yang, Hua Wang

**Affiliations:** 1 Department of Toxicology, School of Public Health, Anhui Medical University, Hefei 230032, China; 2 Key Laboratory of Environmental Toxicology of Anhui Higher Education Institutes, Hefei 230032, China; 3 Department of Medical Genetics and Prenatal Diagnosis, Wuxi Maternity and Child Health Care Hospital, Wuxi 214002, China

**Keywords:** micronutrients, iron, pregnancy, small for gestational age, mild thinness, preschoolers

## Abstract

The supplementation of multiple micronutrients throughout pregnancy can reduce the risk of adverse birth outcomes and various diseases in children. However, the long-term effect of maternal multiple micronutrient levels in the second trimester on the overall development of preschoolers remains unknown. Therefore, 1017 singleton mother–infant pairs and 6-year-old preschoolers were recruited based on the China-Wuxi Birth Cohort Study. Meanwhile, information on the demographic characteristics of pregnant women and preschoolers, maternal copper, calcium, iron, magnesium, and zinc levels in whole blood during the second trimester, and neonatal outcomes, were collected. We aimed to investigate the long-term impact of maternal copper, calcium, iron, magnesium, and zinc levels in the second trimester on mild thinness among 6-year-old preschoolers, and the modifying effect of small for gestational age (SGA), within the Chinese population. Multiple logistic regression models revealed that high-level maternal iron in the second trimester reduced the risk of mild thinness [adjusted OR: 0.46 (95% CI: 0.26, 0.80)] among 6-year-old preschoolers. However, no significant association was found for the remaining four maternal essential metal elements. Additionally, the restricted cubic spline function showed that the risk of mild thinness decreased when maternal iron concentration exceeded 7.47 mmol/L in whole blood during the second trimester. Furthermore, subgroup analysis indicated that the long-term protective effect of high-level maternal iron on mild thinness was only observed in SGA infants. Summarily, high-level maternal iron in the second trimester distinctly lowers the risk of mild thinness among 6-year-old preschoolers, especially in preschoolers with birth outcomes of SGA. Our findings offer evidence for the implementation of iron supplementation in the second trimester as a preventive measure against mild thinness in children.

## 1. Introduction

Thinness, which is considered a marker of malnutrition, is classified into three grades: grade Ⅰ (mild, Z_BMI_ < −1), grade Ⅱ (moderate, Z_BMI_ < −2), and grade Ⅲ (severe, Z_BMI_ < −3) in both children and adults, according to the World Health Organization (WHO) [1,2]. A global total of 52 million children under the age of five were found to have mild thinness, with 17 million of them experiencing severe thinness [3]. The prevalence of mild thinness varied across different regions, including 8.4% in the Philippines, 9% to 13% in China, and 15.5% in sub-Saharan Africa [1,4,5]. In 2012, the member states of the WHO formally endorsed the Global Nutrition Targets, a comprehensive agenda aimed at improving nutrition worldwide by the year 2025, including addressing the issue of thinness among children under the age of five [6]. Furthermore, the Sustainable Development Goal (SDG) 2 set forth the ambitious goal of eliminating all forms of malnutrition by 2030, which could be seen as interconnected with various other child health aspirations outlined in multiple other SDGs [7,8,9]. Hence, the prevalence of mild thinness among infants, children, and adolescents emerges as a significant public health concern on a global scale, and it becomes imperative and time-sensitive to find novel risk factors and implement preventive measures.

Essential metal elements, a subset of micronutrients, play a crucial role in maintaining children’s health. Numerous previous surveys indicated that thinness in childhood may be attributed to multiple micronutrient deficiencies or malnutrition during childhood [10,11,12]. Two randomized controlled trials conducted in Nigeria and Uganda provided evidence that the supplementation of multiple micronutrients in children can effectively prevent malnutrition and thinness [13,14]. The transfer of micronutrients from the mother to the fetus primarily occurs through the placenta [15,16,17]. Besides, the presence of micronutrients in the maternal body during gestation is crucial for embryogenesis, fetal growth, and maternal well-being. Following the developmental origins of health and disease theory, unfavorable conditions experienced during gestation can profoundly impact the onset of diseases in childhood [18]. A recent study indicated that a supplement of calcium was associated with a reduction in systolic blood pressure in childhood [19]. High-level maternal magnesium and copper during pregnancy were related to the low risk of autism spectrum disorder and depression in childhood [20,21]. Additionally, supplements of iron and zinc during pregnancy could improve cognitive function, and iron deficiency during pregnancy may impair cognitive function in childhood [22]. Summarily, many previous researches primarily concentrated on exploring the correlation between levels of various maternal essential metal elements and the development of neurological and cardiovascular diseases in childhood. However, the effect of maternal essential metal elements levels in the second trimester on mild thinness among preschoolers remains unknown.

Fetal growth restriction (FGR) refers to a condition wherein the fetus fails to attain its genetically predetermined growth potential, resulting in small for gestational age (SGA) and low birth weight (LBW) [23,24,25]. Numerous previous studies indicated that FGR is associated with increased neonatal mortality and elevated risk of cardiovascular and metabolic diseases in adulthood [25,26,27]. Multiple epidemiological studies reported a global prevalence of 14.6% for LBW and 27% for SGA in low- and middle-income countries [28,29]. SGA is defined as a birthweight below the 10th percentile for a given gestational age [30]. Reportedly, pregnant women with low-level iron, magnesium, and zinc during pregnancy more often deliver SGA or LBW infants, and increased copper levels in umbilical cord blood were associated with SGA [31,32,33]. Then, infants born with SGA have a higher risk of long-term metabolic diseases and impaired cognition in childhood [34,35]. Additionally, international consensus guidance on SGA indicated that children born with SGA were likely to have persistent short statures at 2–4 years old, and be treated with additional growth hormone [36]. Summarily, abnormal maternal essential metal element levels, including copper, calcium, iron, magnesium, and zinc, may impact the overall growth and development of children through influencing fetal growth. Nevertheless, the long-term impact of maternal essential metal elements during the second trimester on the overall growth and development of preschoolers is unclear, and the modifying effect of SGA remains unexplored.

Wuxi, a prefecture-level city of Jiangsu Province in eastern China, is well known because of its developed economy, relatively high-level informatization of the health service system, and health literacy. Meanwhile, the prevalence of mild thinness in Wuxi was unknown. It is reported that the growth of children in the preschool period is an important developmental life stage that lays the foundation for long-term health outcomes [37]. In China, 6-year-old preschoolers need to participate in medical examinations before enrolling in primary school, and the collection of medical information about them is complete and convenient for us. The second trimester of pregnancy represents a crucial period during which fetal organ development undergoes significant changes. Consequently, we enlisted 1017 singleton mother–infant pairs and 6-year-old preschoolers in Wuxi. The social determinants, such as maternal age and newborn gender, and the health-related factors, such as pre-pregnancy body mass index (BMI), parity, gravidity, and gestational age, which were widely controlled as confounding variables in previous studies with regard to the impact of pregnant exposure characteristics on the development of fetuses and offspring [15,38,39]. Medical information, such as maternal age, pre-pregnancy BMI, parity, gravidity, gestational age, copper, calcium, iron, magnesium, and zinc levels in whole blood during the second trimester, neonatal outcomes, and the information on medical examinations before enrolling in primary school for 6-year-old preschoolers were obtained to establish a retrospective cohort study. Using the available database, we will investigate the long-term impact of maternal copper, calcium, iron, magnesium, and zinc levels in the second trimester on mild thinness among 6-year-old preschoolers, and the modifying effect of SGA, within the Chinese population. Iron deficiency during gestation could frequently cause FGR and anemia in childhood, which impair cardiac function and neurodevelopment in childhood [31,40,41]. Thus, we speculate that high-level maternal iron in the second trimester distinctly lowers the risk of mild thinness among 6-year-old preschoolers, especially in preschoolers with birth outcomes of SGA.

## 2. Methods

### 2.1. The Background of the Cohort

The China-Wuxi Birth Cohort Study (C-WBCS) is a retrospective birth cohort study conducted by the Maternity and Child Health Care Hospital, a Class III Grade I hospital, located in Wuxi, China. The work of collecting information was conducted from 2019 to 2022. The present study obtained information on maternal medical examinations during pregnancy and neonatal outcomes detected and diagnosed by medical workers of Wuxi Maternity and Child Health Care Hospital, from the system of electronic medical records. Demographic characteristics of pre-pregnancy were collected through the System of Healthy Resident Archives, investigated and entered by medical workers of all medical institutions with maternal and child health functions in Wuxi. The Jiangsu Maternal and Child Health Information System, which came into use in 2018, was utilized to collect physical examination data about the development and growth of children, including the information on 6-year-old preschoolers’ medical examinations before enrolling in primary school, input by the medical workers of community hospitals. This database of the retrospective cohort study collected important medical information by matching participants’ information one by one from the aforementioned three components. Frequent data quality checks were performed during data entry, which included double data entries, the elimination of duplicate entries, and checking for the participant’s unique identification number and error entry. The pregnant women who gave birth at Wuxi Maternity and Child Care Hospital between 2012 and 2014 and corresponding 6-year-old preschoolers between 2018 and 2020 were chosen as objects in this study. The objective of this study aimed to illustrate the associations of maternal factors during pregnancy with birth outcomes and the development and growth of children within the Chinese population.

### 2.2. Participants

**Figure 1:**
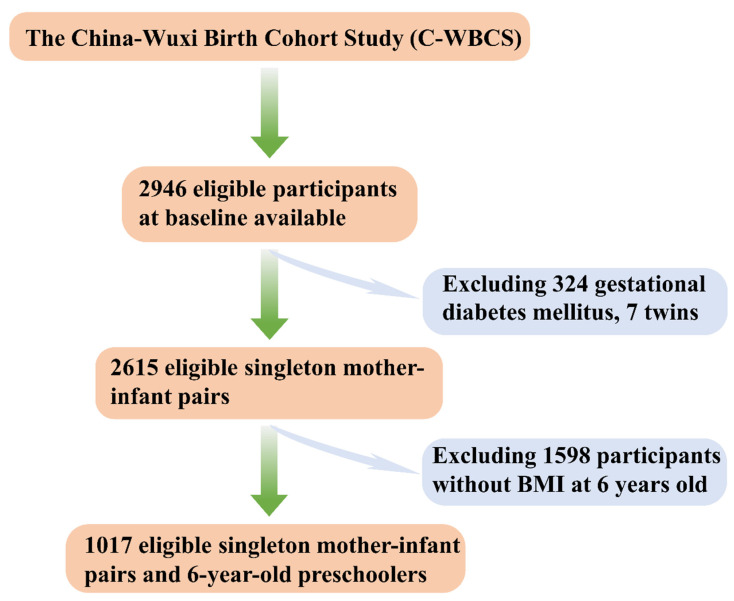
Flow chart of the inclusion of participants in the China-Wuxi Birth Cohort Study (C-WBCS).

Based on C-WBCS, we gathered data about maternal copper, calcium, iron, magnesium, and zinc concentrations in whole blood during the second trimester (spanning from 14 to 27 weeks [42]), as well as the subsequent neonatal outcomes, demographic attributes, and BMI of preschoolers at 6 years old in Wuxi. The criteria for exclusion of participation in the present study were as follows: inability to provide informed consent, mental disorders, gestational diabetes mellitus, heart disease, thyroid-related disease, a history of ≥ 3 previous miscarriages, or plans to leave the Wuxi before delivery. For this study, eligible participants were mother-and-singleton-offspring pairs, with data available for maternal copper, calcium, iron, magnesium, and zinc concentrations in the whole blood during the second trimester and the BMI of 6-year-old preschoolers. As depicted in Figure 1, a total of 2946 pregnant women, aged 18 years or older, who delivered at Wuxi Maternity and Child Health Care Hospital between 2012 and 2014, were initially included in the study. Among them, 324 cases of gestational diabetes mellitus and 7 cases of twin pregnancies were excluded. Subsequently, a cohort in Wuxi of 2615 eligible singleton mother–infant pairs was utilized to investigate the sensitivity and stability of the results that the associations between five maternal essential metal elements levels in the second trimester and the risk of SGA. Furthermore, 1598 participants who lacked information on their BMI at 6 years old were subsequently excluded from the analysis. Ultimately, a total of 1017 eligible singleton mother–infant pairs and 6-year-old preschoolers in Wuxi were utilized to investigate the potential associations of the maternal levels of five essential metal elements during the second trimester with the risk of SGA and mild thinness in preschoolers.

### 2.3. Measurement of Five Essential Metal Elements Concentrations

During the second trimester of the study, all pregnant women were required to provide whole blood samples. Five essential metal element concentrations were then measured using the Graphite Furnace Atomic Absorption Spectrometer (BH5100T, Bohui, Beijing, China). Before analysis, 40 μL of whole blood was mixed with 1200 μL of a dilute solution and left at room temperature for 30 min. The detection process, strictly following the instructions provided in the user’s manual, was carried out by a clinical laboratory technician. The resulting data was subsequently recorded in the medical records of the Wuxi Maternity and Child Health Care Hospital.

### 2.4. Definitions of SGA and LBW

A novel approach for identifying SGA infants was informed by a globally recognized publication in *the Lancet* [43]. Consequently, this approach was employed to establish the fetal intrauterine growth curve and to classify infants as SGA if their birthweight fell below the 10th percentile for their gestational age and gender based on our retrospective cohort study. LBW infants were defined as those weighing less than 2500 g, following the WHO guidelines.

### 2.5. Definitions of Mild Thinness of Preschoolers

According to the WHO, thinness in adults is categorized into three grades: grade Ⅰ (mild, Z_BMI_ < −1), grade Ⅱ (moderate, Z_BMI_ < −2), and grade Ⅲ (severe, Z_BMI_ < −3). A study has suggested that similar cutoffs have been established for children and adolescents based on adult data, thereby providing definitions of thinness across different age groups [2]. Therefore, the WHO child growth standards of BMI-for-age, conducted using the LMS method, define Z_BMI_ < −1 as “mild thinness” in different gender and age.

### 2.6. Definitions of Gestational Diabetes Mellitus

Gestational diabetes mellitus was diagnosed using the 75 g oral glucose tolerance test (OGTT) following the standard set by the International Association for Diabetes in Pregnancy Study Group. The results were categorized based on specific cut-off points, including fasting plasma glucose (FPG, OGTT) ≥ 5.1 mmol/L, 1 h plasma glucose (1 h PG, OGTT1) ≥ 10.0 mmol/L, or 2 h plasma glucose (2 h PG, OGTT2) ≥ 8.5 mmol/L [44].

### 2.7. Statistical Analysis

The determination of the minimum sample size for the present study was achieved through the utilization of a specific formula:

$$N=\frac{Z^{2}\times p\left(1-p\right)}{d^{2}}$$

for 95% confidence intervals *α* = 0.05, *Z*_0.05_ = 1.962, *d* = margin error and *p* = the proportion of disease. A study reported that the prevalence of thinness in children was 9–13% throughout China [5]. When the *p* = 9% and *d* = 2%, the estimated minimal sample size was 788. The present study collected a sample size that was sufficiently large to accurately represent the population with a minimal margin of error. The determination of the sample size was not based on test power without a preliminary study. The normality of the data distribution was evaluated using the Shapiro–Wilk test. For non-normally distributed data, absolute numbers and percentages were used to describe categorical variables. The maternal age index was categorized into two groups based on whether the age was above or below 35 years, as advanced maternal age is typically considered to be 35 years or older. Similarly, the variable for pre-pregnancy BMI was divided into three groups based on the WHO’s definitions of thinness (BMI less than 18.00 kg/m^2^) and overweight (BMI more than 25.00 kg/m^2^). Abnormal pregnancy, encompassing premature and prolonged labor, was defined as gestational ages of delivery less than 37 weeks and more than 42 weeks, respectively. Consequently, the variable of gestational age was divided into three categories: less than 37 weeks, between 37 and 42 weeks, and more than 42 weeks. Gravidity and parity indicators were categorized into two groups: 1 and more than 1. The Chi-square test was adopted for categorical variables to explore associations between basic characteristics and the mild thinness of 6-year-old preschoolers. The percentiles of five essential metal elements levels in whole blood during the second trimester were calculated.

Maternal concentrations of these essential metal elements in whole blood during the second trimester were categorized into three groups based on tertiles, with the first tertile (T1) serving as the reference group when using a multiple logistic regression model. Subsequently, the strength of correlation among multiple indexes was determined using Spearman’s rank correlation, with weak correlation defined as <0.40, moderate correlation as 0.40–0.70, and strong correlation as >0.70 [45]. The multiple logistic regression model using mild thinness of 6-year-old preschoolers and SGA as outcome variables were established. The social determinants, such as maternal age and newborn gender, and health-related factors, such as pre-pregnancy BMI, parity, gravidity, and gestational age, had been chosen for adjustment in model 1 to control confounding factors as much as possible. Additionally, the other four essential metal elements were further adjusted in adjusted model 2. The trends were estimated by treating the tertile as a continuous variable. This study employed mediation models to elucidate the mediating effects of birthweight on the relationships between pre-pregnancy BMI and preschoolers’ BMI. The proportion of the mediated effect was calculated using the formula indirect effect/(indirect effect + direct effect). Additionally, the restricted cubic spline (RCS) function was utilized to estimate the dose–response relationship between maternal iron concentration and the risk of mild thinness among 6-year-old preschoolers. The *p*-value for the overall association and the non-linear association was used to evaluate the overall association and any non-linear association between maternal iron concentrations and the risk of mild thinness in 6-year-old preschoolers. Subgroup analyses were conducted in parity, gravidity, newborn gender, and SGA participants and sensitivity analyses were conducted in 2615 eligible singleton mother–infant pairs to estimate partial associations in the present study. The RCS function and other statistical analysis were carried out in R 4.2.2 using the “rms” package (version 6.7–0) software and SPSS 26.0 statistical software (IBM Corp., Armonk, NY, USA), respectively. All reported *p*-values were calculated using a two-sided Wald test, and *p* < 0.05 was considered to indicate statistical significance.

## 3. Results

### 3.1. Maternal Basic Characteristics and Neonatal Birth Outcomes

**Figure 2:**
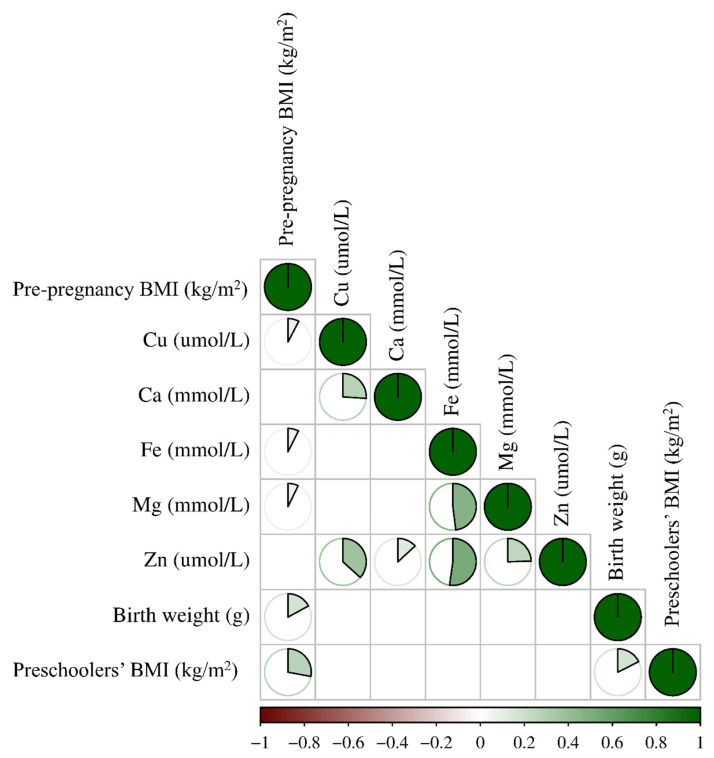
Spearman correlations among multiple indexes. The color depth and the size of the pie indicate the Spearman correlation coefficients. A blank grid presents a *p*-value of more than 0.05. Notes: BMI, body mass index; Cu, copper; Ca, calcium; Fe, iron; Mg, magnesium; Zn, zinc.

In the present study, maternal basic characteristics and neonatal birth outcomes of 1017 singleton preschoolers at the age of 6 were analyzed. Overall, the prevalence of mild thinness, SGA, and LBW among 6-year-old preschoolers were found to be 13.1% (133/1017), 14.1% (143/1017), and 2.0% (20/1017), respectively (Table 1). Table 1 revealed a statistically significant disparity in pre-pregnancy BMI, newborn gender, the prevalence of SGA, and maternal iron levels (*p* < 0.05), however, no significant differences were observed in maternal age, gestational age, gravidity, parity, and prevalence of LBW and the other four maternal essential metal elements (*p* > 0.05) between the groups of preschoolers classified as having mild thinness and non-mild thinness. As presented in Table 2, the median and interquartile range of maternal copper, calcium, iron, magnesium, and zinc concentrations in whole blood during the second trimester were 23.22 (19.87–27.15) μmol/L, 1.68 (1.59–1.78) mmol/L, 7.48 (7.03–7.90) mmol/L, 1.40 (1.31–1.49) mmol/L, and 86.36 (78.08–95.61) μmol/L, respectively. The Spearman correlations among multiple indexes were depicted in Figure 2, revealing that there existed a moderate association between the preschoolers’ BMI and the birthweight of newborns, as well as a weak association between the preschoolers’ BMI and maternal BMI (*p* < 0.05). Additionally, a weak correlation was observed between the birthweight of newborns and maternal BMI (*p* < 0.05). Meanwhile, maternal iron concentrations in whole blood during the second trimester exhibited a weak correlation with maternal BMI (*p* < 0.05).

### 3.2. Association of Five Maternal Essential Metal Elements Levels at the Second Trimester with the Mild Thinness of 6-Year-Old Preschoolers

**Figure 3:**
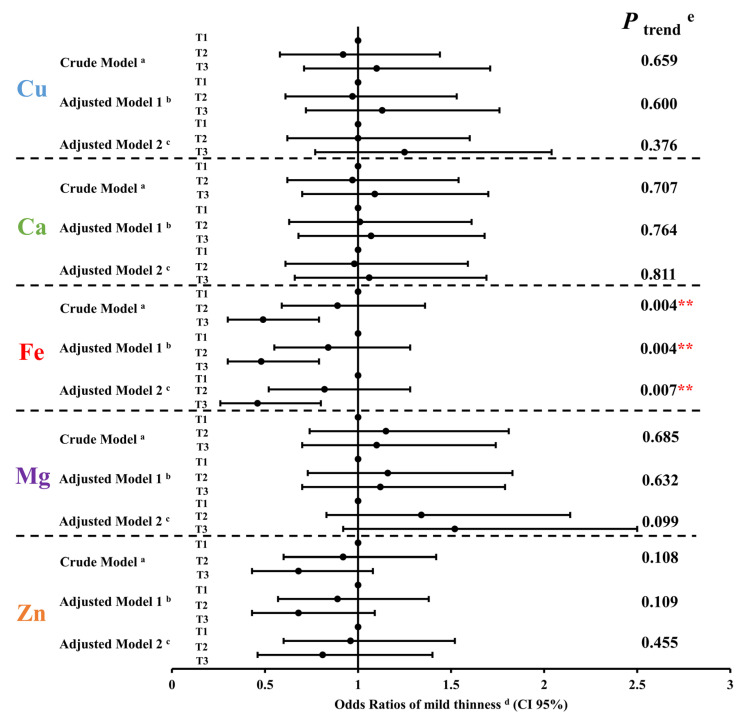
Mild thinness of preschoolers in relation to five maternal essential metal elements levels in the second trimester in the logistic regression model. The odds ratios (OR) and 95% confidence intervals (CIs) were presented for each tertile compared with the third tertile (T1). Notes: Cu, copper; Ca, calcium; Fe, iron; Mg, magnesium; Zn, zinc; T1, 1st tertile; T2, 2nd tertile; T3, 3rd tertile; CI, confidence interval. ^a^ Crude model. ^b^ Adjusted model 1: adjusted for maternal age, pre-pregnancy BMI, parity, gravidity, newborn gender, and gestation age. ^c^ Adjusted model 2: adjusted for variables in model 1 and the other four maternal essential metal elements. ^d^ Mild thinness is defined as body mass index z-score < −1 for 6-year-old preschoolers of different genders. ^e^ *p* trend was the *p* value for the trend. ** *p* <0.01.

Multiple logistic regression models were used to investigate the associations between the maternal levels of five essential metal elements in whole blood during the second trimester and the risk of mild thinness in 6-year-old preschoolers. As shown in Figure 3, when compared to the pregnant women with the first tertile of iron level, their descendants, compared to those with the third tertile of iron level, were less likely to be mildly thin at 6 years old: OR = 0.49, 95% CI: 0.30, 0.79; OR = 0.48, 95% CI: 0.30, 0.79; OR = 0.46, 95% CI: 0.26, 0.80 in the crude model, adjusted model 1 (adjusted for maternal age, pre-pregnancy BMI, parity, gravidity, newborn gender, and gestation age) and adjusted model 2 (adjusted for variables in model 1 and the other four maternal essential metal elements), respectively. Besides, a distinct inverse correlation was observed between maternal iron levels during the second trimester and the odds ratio of mild thinness among 6-year-old preschoolers in adjusted model 2 [OR (95% CI): T1 = 1.00 (reference); T2 = 0.82 (0.52, 1.28); T3 = 0.46 (0.26, 0.80); *p* = 0.007 for trend]. No significant correlations were found between the levels of the remaining four essential maternal metal elements and mild thinness in 6-year-old preschoolers.

### 3.3. Dose–Response Relationship between Maternal Iron Concentrations in the Second Trimester and Mild Thinness of 6-Year-Old Preschoolers

**Figure 4:**
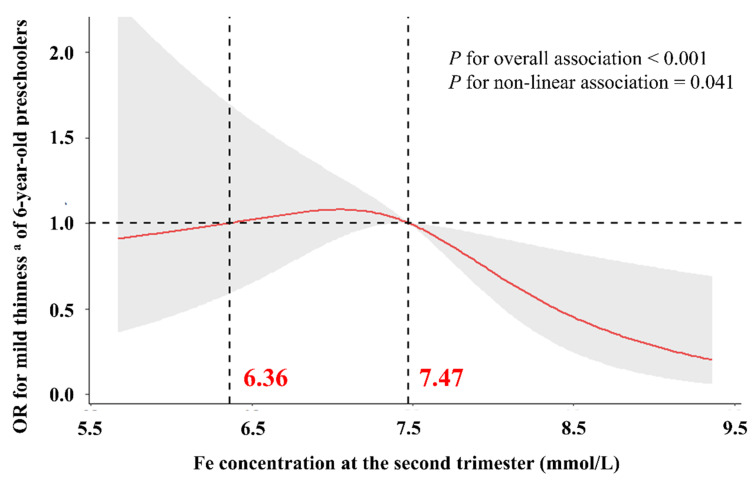
Dose–response relationship between maternal iron concentrations in the second trimester and mild thinness of 6-year-old preschoolers. The associations were estimated using restricted cubic spline functions with three knots. Models were adjusted for maternal age, pre-pregnancy BMI, parity, gravidity, newborn gender, gestation age, and the other four maternal essential metal elements. The light grey area represents the 95% CIs. Two vertical black lines represent iron concentrations when the OR for mild thinness of 6-year-old preschoolers was 1.0. The horizontal black line represents when the OR for mild thinness of 6-year-old preschoolers was 1.0. The abscissa of the intersection of the red line (the curve of RCS function) and the horizontal black line were written in red. Notes: OR, odds ratios; Fe, iron. ^a^ Mild thinness is defined as body mass index z-score < −1 for 6-year-old preschoolers of a different gender.

The RCS function with three knots was further applied to evaluate the dose–response relationships between maternal iron concentrations in whole blood during the second trimester and mild thinness of 6-year-old preschoolers. As depicted in Figure 4, a statistically significant non-linear dose–response relationship curve was fitted between maternal iron concentrations in the second trimester and the risk of mild thinness in 6-year-old preschoolers (*p*-value for overall association < 0.001 and non-linear association = 0.041). The risk of mild thinness among 6-year-old preschoolers exhibited relative stability and proximity to 1.0 when the maternal iron concentration in whole blood during the second trimester was below 7.47 mmol/L. Conversely, a noteworthy decline in the risk of mild thinness among 6-year-old preschoolers was observed when the maternal iron concentration in whole blood during the second trimester exceeded 7.47 mmol/L.

### 3.4. Relationship between Five Maternal Essential Metal Elements Levels at the Second Trimester and Small for Gestational Age

**Figure 5:**
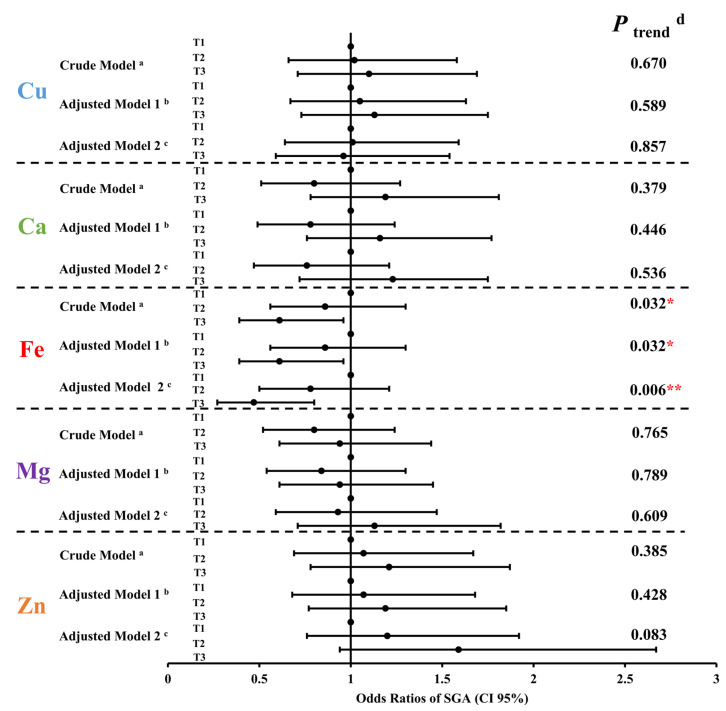
Small for gestational age in relation to five maternal essential metal elements levels in the second trimester using logistic regression analysis. The odds ratios (OR) and 95% confidence intervals (CIs) were presented for each tertile compared with the third tertile (T1). Notes: Cu, copper; Ca, calcium; Fe, iron; Mg, magnesium; Zn, zinc; T1, 1st tertile; T2, 2nd tertile; T3, 3rd tertile; SGA, small for gestational age; CI, confidence interval. ^a^ Crude model. ^b^ Adjusted model 1: adjusted for maternal age, pre-pregnancy BMI, parity, gravidity, newborn gender, and gestation age. ^c^ Adjusted model 2: adjusted for variables in model 1 and the other four maternal essential metal elements. ^d^ *p* trend was the *p* value for the trend. ** *p* <0.01, * *p* <0.05.

The results presented in Table 1 indicate a significant disparity in the prevalence of SGA between the mild thinness and non-mild thinness groups (*p* = 0.013). Thus, the multiple logistic regression model was used to analyze the associations between five maternal essential metal elements levels in whole blood during the second trimester and the odds ratios of SGA. When compared to the pregnant women with the first tertile of maternal iron level, those with the third tertile of maternal iron level were less likely to have SGA infants: OR = 0.61, 95% CI: 0.39, 0.96; OR = 0.61, 95% CI: 0.39, 0.96; OR = 0.47, 95% CI: 0.27, 0.80 in the three different models, respectively (Figure 5). In addition, a negative relationship was noted between maternal iron levels in the second trimester and the OR of SGA in adjusted model 2 [OR (95% CI): T1 = 1.0 (reference); T2 = 0.78 (0.50, 1.21); T3 = 0.47 (0.27, 0.80); *p* = 0.006 for trend]. Concerning the other four essential metal elements, there were no obvious relationships between their levels and SGA.

### 3.5. Association between Small for Gestational Age and Mild Thinness of 6-Year-Old Preschoolers

The relationship between SGA and the mild thinness of 6-year-old preschoolers was evaluated using multiple logistic regression models. As presented in Table 3, the risk for SGA infants progressing to mild thinness 6-year-old preschoolers in comparison to non-SGA infants were 1.78 (1.12, 2.83), 1.80 (1.12, 2.89), and 1.72 (1.07, 2.78) times in the three different models.

### 3.6. Subgroup and Sensitivity Analysis

Subgroup analysis was adopted to assess the sensitivity of different populations. In Table 4, it was observed that maternal high-iron levels in whole blood during the second trimester significantly reduced the odds ratio of mild thinness in 6-year-old preschoolers who were SGA infants [OR = 0.37 (0.19–0.69); OR = 0.29 (0.14–0.58); OR = 0.34 (0.14–0.87), *p* for interaction = 0.034, 0.026, 0.026 in the three different models]. To validate the robustness of the above findings, a larger population consisting of 2615 eligible singleton mother–infant pairs was explored, and the primary results remained stable and reliable. Details of the summary results are presented in Supplementary Tables S1–S3 and Supplementary Figure S1.

## 4. Discussion

In developing countries, the prevalence of moderate and severe stunting and underweight was 29.9% and 19.4% in 2011 and the chance of meeting Millennium Development Goal 1, including an indication that the proportion of children younger than 5 years who were underweight was less than 5% in general, was negligible [5]. There was a great difference in the prevalence of moderate stunting and underweight globally in 2011. Countries such as Chile had as low as nearly 1–2% incidence of moderate stunting and underweight, but Timor-Leste had as high as nearly 40–55% [5]. The present study examined the prevalence of mild thinness among preschoolers in Eastern China. The findings revealed that the prevalence of 6-year-old preschoolers’ mild thinness was 13.1% (133/1017), which aligns with a previous study indicating that mild thinness affects approximately 9–13% of children across China [5]. Globally, one in five (23.4 million) livebirths were small for gestational age in 2020, especially in southern Asia [46]. Meanwhile, the present study further assessed the prevalence of SGA using a defined method published in *the Lancet*. Our finding demonstrated that the occurrence rate of SGA was 14.1% (143/1017) in Eastern China, which followed the previous studies revealing that the prevalence of SGA was 13.8% in Europe and that was 13.4% in Northwestern China [47,48].

Also, the present study investigated five maternal essential metal element levels in whole blood during the second trimester among a cohort of 1017 eligible singleton preschoolers at 6 years old in Eastern China, using a retrospective birth cohort design. The results indicated that the median and interquartile range of maternal Fe, Cu, and Zn levels in whole blood during the second trimester for all participants were 7.48 mmol/L (7.03 mmol/L–7.90 mmol/L), 23.22 μmol/L (19.87 μmol/L–27.15 μmol/L), and 86.36 μmol/L (78.08 μmol/L–95.61 μmol/L), respectively. Similarly, a case–control study conducted in Central China demonstrated that the mean of maternal Fe level in whole blood during pregnancy was 7.14 mmol/L [49]. The PROTECT birth cohort study conducted in Puerto Rico displayed that the median and interquartile range of maternal Cu and Zn levels in whole blood during pregnancy were 25.45 μmol/L (22.49 μmol/L–28.02 μmol/L) and 73.54 μmol/L (65.35 μmol/L–81.75μmol/L) [50]. Another nested case–control study performed in Northern China showed that the mean of maternal Zn level in whole blood was 100.46 ± 30.77 μmol/L (± SD) [51]. The data indicate that the levels of Fe, Cu, and Zn in pregnant women from various regions align with our findings.

Our findings implied a notable relationship between high-level maternal iron in whole blood during the second trimester and a notably decreased risk of mild thinness among their 6-year-old descendants. Similarly, numerous epidemiological investigations elucidate that maternal iron supplements during gestation can mitigate the risk of various diseases affecting children’s nervous systems, respiratory systems, chronic metabolic systems, and skeletal systems [52,53,54,55,56,57,58]. Additionally, it was found that a cut-off value of maternal iron level in whole blood during the second trimester, exceeding 7.47 mmol/L in Wuxi, was associated with a significant reduction in the risk of mild thinness among 6-year-old preschoolers. Furthermore, it implied that the concentration of iron in the whole blood during the second trimester should reach at least 7.47 mmol/L in Wuxi if we want to prevent mild thinness in children through the supplementation of iron. The median of maternal iron concentration was 7.48 mmol/L in our cohort, which implied that in approximately half of their descendants existed the risk of experiencing mild thinness at 6 years old in Wuxi. Therefore, our findings offer empirical support and clinical recommendations for the implementation of iron supplementation during pregnancy as a preventive measure against mild thinness in children. That could provide a theoretical basis and scientific significance for the government to establish standards for iron supplementation during pregnancy to prevent mild thinness in children and preferably meet the Healthy China strategy, Millennium Development Goal (MDG) 1, and multiple SDGs.

Furthermore, the present study illustrated that high-level maternal iron was related to a significant decrease in the risk of SGA. This finding was in line with a systematic review that indicated a heightened risk of FGR was associated with maternal iron deficiency during pregnancy [31]. Additionally, two extensive prospective studies conducted in China reported an increased risk of SGA linked to maternal iron deficiency, anemia, and malnutrition during pregnancy [48,59]. Previous animal experiments also indicated that inadequate maternal iron intake during gestation impaired placental and fetal development through altering placental trophoblast differentiation in rats [60]. Meanwhile, it was observed that SGA infants were more prone to mild thinness at 6 years old. These findings collectively suggest that SGA may be a crucial factor in the relationship between high-level maternal iron in whole blood during the second trimester and a significant reduction in the risk of mild thinness among 6-year-old preschoolers. Therefore, pregnant women with a high probability of delivering SGA infants should been paid more attention by people when the government established standards for iron supplementation during pregnancy to prevent mild thinness in children.

Iron, a kind of essential metal element, is required for numerous crucial enzymatic reactions, placental development, and fetal growth [61,62]. Prior research has demonstrated that maternal iron insufficiency during pregnancy may lead to the apoptosis of placentae and fetal cells via the triggering of oxidative stress, ultimately resulting in SGA [63,64,65,66]. Fibroblast growth factor-23 (FGF23), functioning as a hormone that regulates vitamin D, primarily maintains the balance of phosphate within the body by increasing the excretion of phosphate through urine. A secondary analysis of a randomized controlled trial displayed that maternal iron supplements during gestation had the potential to decrease total FGF23 concentrations in both mothers and neonates [58]. Insulin-like growth factor 1 (IGF-1), a member of the tyrosine-kinase receptor family, plays a crucial role in the growth and development of multiple organs and cells, such as the musculoskeletal system [67,68,69]. Another randomized controlled trial demonstrated that maternal iron supplementation during gestation may lead to a reduction in IGF-1 levels in children [54]. In conclusion, the potential mechanisms underlying the correlation between high-level maternal iron in whole blood during the second trimester and a significant reduction in the risk of mild thinness among 6-year-old preschoolers may involve IGF-1, FGF23, and oxidative stress.

Our study possesses several strengths. Firstly, it involves the long-term monitoring of children and the compilation of medical records from a Class III Grade I hospital, enabling the establishment of a retrospective birth cohort. Secondly, we employ an innovative analysis technique known as the RCS function to examine the dose–effect relationship between maternal iron levels in whole blood during the second trimester and the risk of mild thinness among 6-year-old preschoolers. Thirdly, we take into account the potential effects of multiple concomitant variables, such as copper, calcium, magnesium, and zinc. Lastly, our findings offer empirical support and clinical recommendations for the implementation of iron supplementation during pregnancy as a preventive measure against mild thinness in children and contribute to meeting the Healthy China strategy, MDG 1, and multiple SDGs.

Of course, the present study also has several limitations. Firstly, other potential environmental confounders, such as heavy metals, microplastics, PM_2.5,_ and other micronutrients, were not controlled [70,71,72,73,74]. Secondly, potential genetics, lifestyle, and psychological confounders, such as children’s genetic predisposition, eating habits, and sociopsychological predisposition were not controlled [75,76,77,78]. Thirdly, the mechanisms behind the associations were not clarified in our study. Lastly, the critical window in which high-level maternal iron reduces mild thinness of preschoolers at 6 years old and SGA was not evaluated due to a single collection of blood samples during the second trimester. Thus, multicentric large-scale prospective cohort studies with a more robust database are required to further determine the impact of high-level maternal iron in whole blood during the second trimester on the risk of mild thinness at 6 years old, and the modifying effect of SGA, as well as animal experiments to clarify the mechanism behind it in the future.

## 5. Conclusions

High-level maternal iron in whole blood during the second trimester distinctly lowers the risk of mild thinness among 6-year-old preschoolers, maybe primarily by reducing the risk of SGA. No significant association is observed between maternal essential metal element levels and mild thinness among 6-year-old preschoolers. Our findings offer empirical support and clinical recommendations for the implementation of iron supplementation during pregnancy as a preventive measure against mild thinness in children. However, multicentric large-scale prospective cohort studies and animal experiments are required to confirm the above associations and elucidate their mechanism in the future.

## Figures and Tables

**Table 1:** Maternal basic characteristics and neonatal birth outcomes.

Characteristics	Total	Mild Thinness ^a^ (*n* = 133)	Non-Mild Thinness (*n* = 884)	*p*-Value
**Maters**				
Maternal age [years, *n* (%)]				
<36	1010 (99.3)	133 (100.0)	877 (99.2)	0.303
≥36	7 (0.7)	0 (0)	7 (0.8)	
Pre-pregnancy BMI [kg/m^2^, *n* (%)]				
<18.5	171 (16.8)	38 (28.6)	133 (15.0)	<0.001 ***
18.5–25.0	793 (78.0)	93 (69.9)	700 (79.2)	
≥25.0	53 (5.2)	2 (1.5)	51 (5.8)	
Gestational age [weeks, *n* (%)]				
<37	23 (2.3)	4 (3.0)	19 (2.1)	0.710
37–42	972 (95.6)	127 (95.5)	845 (95.6)	
≥42	22 (2.1)	2 (1.5)	20 (2.3)	
Parity [*n* (%)]				
1	928 (91.2)	125 (94.0)	803 (90.8)	0.231
≥2	89 (8.8)	8 (6.0)	81 (9.2)	
Gravidity [*n* (%)]				
1	672 (66.1)	84 (63.2)	588 (66.5)	0.446
≥2	345 (33.9)	49 (36.8)	296 (33.5)	
Cu (μmol/L)				
1st tertile (<21.09)	338 (33.3)	44 (33.1)	294 (33.3)	0.719
2nd tertile (21.09–25.63)	340 (33.4)	41 (30.8)	299 (33.8)	
3rd tertile (≥25.63)	339 (33.3)	48 (36.1)	291 (32.9)	
Ca (mmol/L)				
1st tertile (<1.62)	319 (31.4)	41 (30.8)	278 (31.5)	0.875
2nd tertile (1.62–1.74)	343 (33.7)	43 (32.3)	300 (33.9)	
3rd tertile (≥1.74)	355 (34.9)	49 (36.9)	306 (34.6)	
Fe (mmol/L)				
1st tertile (<7.19)	336 (33.1)	54 (40.6)	282 (31.9)	0.009 **
2nd tertile (7.19–7.77)	342 (33.6)	50 (37.6)	292 (33.0)	
3rd tertile (≥7.77)	339 (33.3)	29 (21.8)	310 (35.1)	
Mg (mmol/L)				
1st tertile (<1.33)	321 (31.6)	39 (29.3)	282 (31.9)	0.819
2nd tertile (1.33–1.46)	356 (35.0)	49 (36.9)	307 (34.7)	
3rd tertile (≥1.46)	340 (33.4)	45 (33.8)	295 (33.4)	
Zn (μmol/L)				
1st tertile (<80.60)	338 (33.3)	50 (37.6)	288 (32.6)	0.241
2nd tertile (80.60–92.15)	340 (33.4)	47 (35.3)	293 (33.1)	
3rd tertile (≥92.15)	339 (33.3)	36 (27.1)	303 (34.3)	
**Infants**				
Newborn gender [*n* (%)]				
Male	535 (52.6)	57 (42.9)	478 (54.1)	0.016 *
Female	482 (47.4)	76 (57.1)	406 (45.9)	
SGA [*n* (%)]				
Yes	143 (14.1)	28 (21.1)	115 (13.0)	0.013 *
No	874 (85.9)	105 (78.9)	769 (87.0)	
LBW [*n* (%)]				
Yes	20 (2.0)	2 (1.5)	18 (2.0)	0.680
No	997 (98.0)	131 (98.5)	866 (98.0)	

Notes: BMI, body mass index; SGA, small for gestational age; LBW, low birth weight; Cu, copper; Ca, calcium; Fe, iron; Mg, magnesium; Zn, zinc. ^a^ Mild thinness is defined as a body mass index z-score < −1 for 6-year-old preschoolers of different genders. *** *p* < 0.001, ** *p* < 0.01, * *p* < 0.05.

**Table 2:** The distribution of five maternal essential metal element concentrations in the second trimester.

Metal Elements	Percentile							
	Min	5	25	50	75	95	Max	IQR
Cu (μmol/L)	11.86	15.90	19.87	23.22	27.15	32.73	40.10	7.28
Ca (mmol/L)	1.18	1.47	1.59	1.68	1.78	1.96	2.13	0.19
Fe (mmol/L)	4.59	6.24	7.03	7.48	7.90	8.70	11.55	0.87
Mg (mmol/L)	1.13	1.20	1.31	1.40	1.49	1.65	1.91	0.18
Zn (μmol/L)	52.08	66.75	78.08	86.36	95.61	109.75	146.50	17.53

Notes: Min, minimum; Max, maximum; IQR, inter quartile range; Cu, copper; Ca, calcium; Fe, iron; Mg, magnesium; Zn, zinc.

**Table 3:** Association between small for gestational age and mild thinness of 6-year-old preschoolers in the logistic regression model.

	BMI of Preschoolers		Crude Model ^a^	*p*-Value	Adjusted Model 1 ^b^	*p*-Value	Adjusted Model 2 ^c^	*p*-Value
	Mild Thinness ^d^	Non-Mild Thinness	OR (95% CI)		OR (95% CI)		OR (95% CI)	
SGA	28 (21.1)	115 (13.0)	1.78 (1.12–2.83)	0.014 *	1.80 (1.12–2.89)	0.014 *	1.72 (1.07–2.78)	0.026 *
Non-SGA	105 (78.9)	769 (87.0)						

Notes: BMI, body mass index; SGA, small for gestational age; OR, odd ratio; CI, confidence interval; ^a^ Crude model. ^b^ Adjusted model 1: adjusted for maternal age, pre-pregnancy BMI, parity, gravidity, newborn gender, and gestation age. ^c^ Adjusted model 2: adjusted for variables in model 1 and five maternal essential metal elements. ^d^ Mild thinness is defined as body mass index z-score < −1 for 6-year-old preschoolers of different genders. * *p* < 0.05.

**Table 4:** Association between maternal iron level in the second trimester and mild thinness of preschoolers according to subgroups.

Subgroups	Crude Model ^a^	*P* _interaction_	Adjusted Model 1 ^b^	*P* _interaction_	Adjusted Model 2 ^c^	*P* _interaction_
	OR (95% CI)		OR (95% CI)		OR (95% CI)	
**Maters**						
Parity						
1	0.69 (0.55–0.88)	0.325	0.69 (0.54–0.88)	0.297	0.68 (0.52–0.90)	0.280
≥2	1.02 (0.42–2.49)		1.14 (0.44–2.99)		0.64 (0.16–2.49)	
Gravidity						
1	0.76 (0.57–1.00)	0.618	0.76 (0.57–1.02)	0.604	0.75 (0.54–1.05)	0.672
≥2	0.64 (0.44–0.95)		0.63 (0.42–0.95)		0.56 (0.34–0.93)	
**Infants**						
Newborn gender						
Male	0.71 (0.50–0.99)	0.684	0.81 (0.54–1.20)	0.652	1.06 (0.62–1.80)	0.631
Female	0.72 (0.52–0.98)		0.71 (0.50–1.00)		0.66 (0.45–0.96)	
SGA						
Yes	0.37 (0.19–0.69)	0.034 *	0.29 (0.14–0.58)	0.026 *	0.34 (0.14–0.87)	0.026 *
No	0.83 (0.64–1.06)		0.83 (0.64–1.07)		0.76 (0.56–1.02)	

Notes: OR, odd ratio; CI, confidence interval; SGA, small for gestational age. ^a^ Crude model. ^b^ Adjusted model 1: adjusted for maternal age, pre-pregnancy BMI, parity, gravidity, newborn gender, and gestation age. ^c^ Adjusted model 2: adjusted for variables in model 1 and the other four maternal essential metal elements. * *p* <0.05.

## Data Availability

The data presented in this study are available on request from the corresponding author.

## Supplementary Materials

The following supporting information can be downloaded at: https://www.mdpi.com/article/10.3390/nu15183939/s1, Table S1: Basic characteristics of 2615 eligible singleton mother–infant pairs; Table S2: The distribution of maternal essential metal elements concentrations in the second trimester for 2615 eligible singleton mother–infant pairs; Table S3: Small for gestational age in relation to five maternal essential metal elements levels in the second trimester using logistic regression analysis for 2615 eligible singleton mother–infant pairs; Figure S1; Spearman correlations among multiple indexes for 2615 eligible singleton mother–infant pairs.
