# Supplementary material for: The Long-Term Effect of Maternal Iron Levels in the Second Trimester on Mild Thinness among Preschoolers: The Modifying Effect of Small for Gestational Age
Source: Nutrients. 2023 Sep 11;15(18):3939. doi: 10.3390/nu15183939 (PMC10535896; doi:10.3390/nu15183939)
Supplement: Supplementary file 1 [file nutrients-15-03939-s001.zip › nutrients-2576083-supplementary.pdf]

Table S1. Basic characteristics at the second trimester of 2615 eligible singleton mother-infant pairs.

Characteristics	Total	SGA (*n*=374)	Non-SGA (*n*=2241)	*P*-value
**Maters**				
Maternity Age [years, *n*(%)]				
<36	2603 (99.5)	372 (99.5)	2231 (99.6)	0.815
≥36	12 (0.5)	2 (0.5)	10 (0.4)	
Maternity BMI [kg/m^2, *n*(%)]				
<18.5	421 (16.1)	74 (19.8)	347 (15.5)	
18.5-25.0	2050 (78.4)	288 (77.0)	1762 (78.6)	0.019 *
≥25.0	144 (5.5)	12 (3.2)	132 (5.9)	
Gestational age [weeks, *n*(%)]				
<37	61 (2.2)	13 (3.5)	48 (2.1)	
37-42	2480 (94.8)	336 (89.8)	2144 (95.7)	<0.001 ***
≥42	74 (2.8)	25 (6.7)	49 (2.2)	
Parity [*n*(%)]				
1	2392 (91.5)	348 (93.0)	2044 (91.2)	0.239
≥2	223 (8.5)	26 (7.0)	197 (8.8)	
Gravidity [*n*(%)]				
1	1729 (66.1)	274 (73.3)	1455 (64.9)	0.002 **
≥2	886 (33.9)	100 (26.7)	786 (35.1)	
**Infants**				
Newborn gender [*n*(%)]				
Male	1389 (53.1)	188 (50.3)	1201 (53.6)	0.233
Female	1226 (46.9)	186 (49.7)	1040 (46.4)	

Notes: BMI, body mass index; SGA, small for gestational age;

*** P <0.001, ** P <0.01, * P <0.05

Table S2. The distribution of maternal essential metal elements concentrations at the second trimester for 2615 eligible singleton mother-infant pairs.

Metal elements	Percentile							
	Min	5	25	50	75	95	Max	IQR
Cu (μmol/L)	11.30	16.00	20.42	24.00	27.92	33.71	40.10	7.5
Ca (mmol/L)	1.18	1.47	1.59	1.67	1.77	1.95	2.18	0.18
Fe (mmol/L)	4.59	6.21	6.97	7.48	8.00	8.80	11.55	1.03
Mg (mmol/L)	1.12	1.18	1.29	1.38	1.48	1.66	2.02	0.19
Zn (μmol/L)	47.08	68.13	79.15	87.93	97.18	111.69	159.77	18.03

Notes: Min, minimum; Max, maximum; IQR, inter quartile range; Cu, copper; Ca, calcium; Fe, iron; Mg, magnesium; Zn, zinc

Table S3. Small for gestational age in relation to tertile maternal essential metal elements concentrations at the second trimester using logistic regression analysis for 1017 eligible singleton preschoolers at 6 years old.

**Metal elements**	**SGA**	**Non-SGA**	Crude Model [a]	*P*-value	Adjusted Model 1 [b]	*P*-value	Adjusted Model 2 [c]	*P*-value
			OR (95% CI)		OR (95% CI)		OR (95% CI)	
Cu (μmol/L)								
1st tertile (<21.69)	126 (33.7)	745 (33.2)	1.00	-	1.00	-	1.00	-
2nd tertile (21.69-26.62)	139 (37.2)	733 (32.7)	1.12 (0.86-1.46)	0.392	1.17 (0.90-1.53)	0.238	1.13 (0.86-1.48)	0.385
3rd tertile (≥26.62)	109 (29.1)	763 (34.1)	0.84 (0.64-1.11)	0.230	0.87 (0.66-1.15)	0.317	0.79 (0.58-1.07)	0.122
P for trend			0.241		0.334		0.137	
Ca (mmol/L)								
1st tertile (<1.62)	132 (35.3)	725 (32.3)	1.00	-	1.00	-	1.00	-
2nd tertile (1.62-1.74)	119 (31.8)	737 (32.9)	0.89 (0.68-1.16)	0.380	0.89 (0.68-1.16)	0.381	0.88 (0.67-1.15)	0.344
3rd tertile (≥1.74)	123 (32.9)	779 (34.8)	0.87 (0.66-1.13)	0.293	0.86 (0.66-1.12)	0.265	0.89 (0.67-1.17)	0.401
P for trend			0.293		0.265		0.398	
Fe (mmol/L)								
1st tertile (<7.16)	143 (38.2)	724 (32.3)	1.00	-	1.00	-	1.00	-
2nd tertile (7.16-7.80)	127 (34.0)	743 (33.2)	0.86 (0.67-1.12)	0.276	0.84 (0.64-1.09)	0.194	0.73 (0.55-0.97)	0.028 *
3rd tertile (≥7.80)	104 (27.8)	774 (34.5)	0.68 (0.52-0.89)	0.006**	0.69 (0.52-0.91)	0.008 **	0.55 (0.39-0.77)	<0.001 ***
P for trend			0.006 **		0.008 **		<0.001 ***	
Mg (mmol/L)								
1st tertile (<1.32)	115 (30.8)	691 (30.8)	1.00	-	1.00	-	1.00	-
2nd tertile (1.32-1.45)	143 (38.2)	791 (35.3)	1.09 (0.83-1.42)	0.542	1.13 (0.86-1.48)	0.371	1.24 (0.94-1.64)	0.128
3rd tertile (≥1.45)	116 (31.0)	759 (33.9)	0.92 (0.70-1.21)	0.548	0.94 (0.71-1.24)	0.654	1.13 (0.82-1.55)	0.468

P for trend			0.537		0.643		0.444	
Zn (μmol/L)								

Table S3. Cont.

1st tertile (<82.23)	117 (31.3)	753 (33.6)	1.00	-	1.00	-	1.00	-
2nd tertile (82.23-93.90)	134 (35.8)	738 (32.9)	1.17 (0.89-1.53)	0.255	1.17 (0.89-1.53)	0.257	1.33 (1.00-1.77)	0.047 *
3rd tertile (≥93.90)	123 (32.9)	750 (33.5c)	1.06 (0.80-1.39)	0.698	1.05 (0.80-1.39)	0.714	1.47 (1.06-2.04)	0.023 *
P for trend			0.703		0.720		0.020 *	

Notes: SGA, small for gestational age; OR, odd ratio; CI, confidence interval; Cu, copper; Ca, calcium; Fe, iron; Mg, magnesium; Zn, zinc

[a] Crude model.

[b] Adjusted model 1: adjusted for maternity age, maternity BMI, parity, gravidity, newborn gender, gestation age.

[c] Adjusted model 2: adjusted for variables in model 1 and the other four maternal essential metal elements.

*** *P* <0.001, ** *P* <0.01, * *P* <0.05

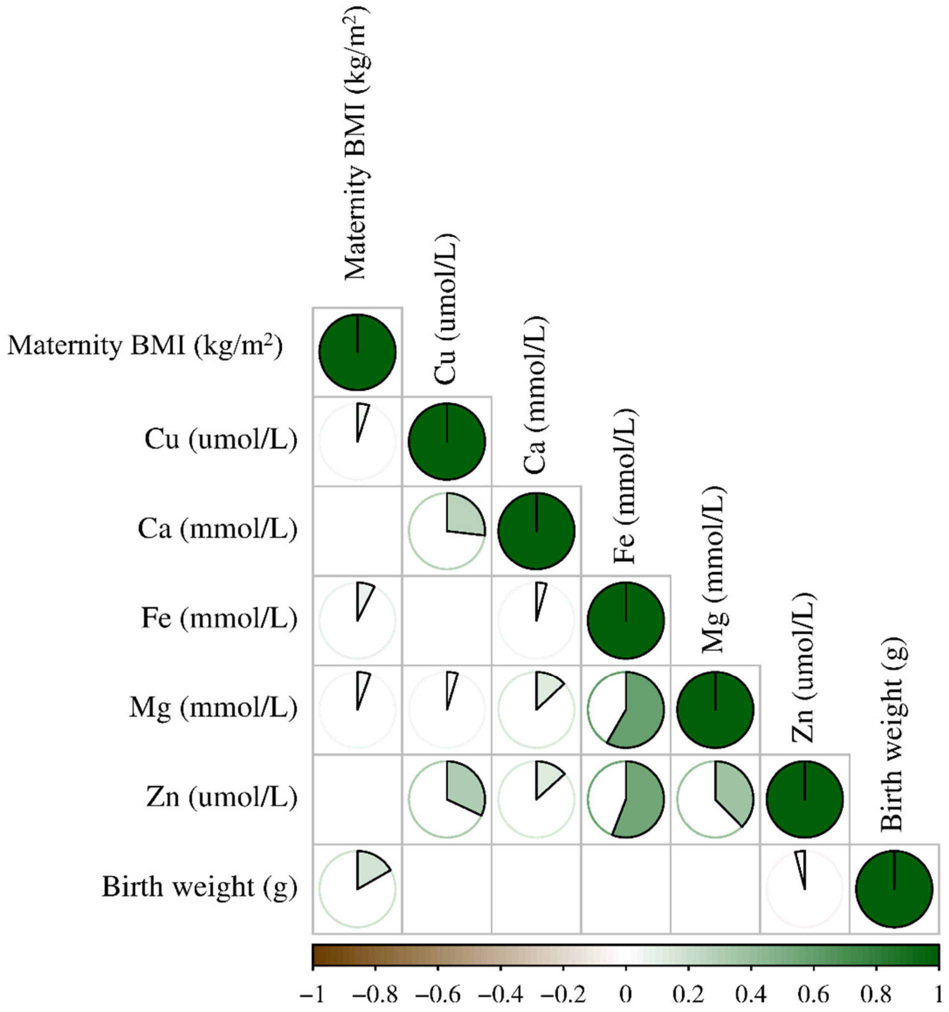

Figure S1. Spearman correlations among multiple indexes for 2615 eligible singleton mother-infant pairs. The color depth and the size of the pie indicates the spearman correlation coefficients. Blank gird presents the *p*-value less than 0.05.
